# Contrast-enhanced Ultrasound Features of Renal Hemangiomas: A Retrospective Descriptive Study

**DOI:** 10.2174/0115734056426319260403204532

**Published:** 2026-04-21

**Authors:** Chunhong Yan, Siying Zhang, Mengna He, Jie Qiu, Jianjian Xiang, Tianan Jiang

**Affiliations:** 1 Department of Ultrasound, First Affiliated Hospital, School of Medicine, Zhejiang University, Hangzhou, China; 2 Department of Radiology, First Affiliated Hospital, School of Medicine, Zhejiang University, Hangzhou, China; 3 Zhejiang University Cancer Center, Zhejiang, Hangzhou, China

**Keywords:** Ultrasound, Renal hemangiomas, Contrast-enhanced ultrasound, Renal mass, Imaging diagnosis, Benign tumor

## Abstract

**Objective:**

This study aimed to investigate the contrast-enhanced ultrasound (CEUS) imaging features of renal hemangiomas and to evaluate their potential role in improving preoperative diagnosis and differential diagnosis.

**Methods:**

In this retrospective study, clinical and ultrasound data from 20 patients with surgically confirmed renal hemangiomas (22 lesions) were analyzed. All patients underwent preoperative conventional ultrasound. Among them, 6 patients (7 lesions) additionally underwent CEUS examination within one month before surgery. Standardized ultrasound techniques and equipment were employed, with focused analysis on the enhancement patterns and hemodynamic characteristics observed on CEUS.

**Results:**

The cohort comprised 10 men and 10 women (mean age 50 years). Most lesions (13/22) were located in the renal medulla. On conventional ultrasound, lesions typically appeared as well-defined, round, hypoechoic nodules, with most showing no significant internal flow on color Doppler imaging. In the 6 patients who underwent CEUS, a characteristic pattern of peripheral nodular enhancement in the arterial phase, followed by progressive centripetal filling, was observed. Peak enhancement intensity was generally comparable to that of the surrounding renal parenchyma. Pathologically, anastomosing hemangioma and capillary hemangioma were the most common subtypes (9 cases each), with immunohistochemical profiles (CD31/CD34 positive, low Ki-67) consistent with benign behavior.

This study reveals a preliminary CEUS pattern of renal hemangiomas characterized by peripheral nodular enhancement with progressive centripetal filling, which may aid in differentiating these rare benign tumors from renal cell carcinoma and help avoid unnecessary surgery. The small sample size is a major limitation, and these findings require validation in larger prospective studies.

**Discussion:**

This study reveals a preliminary CEUS pattern of renal hemangiomas characterized by peripheral nodular enhancement with progressive centripetal filling, which may aid in differentiating these rare benign tumors from renal cell carcinoma, potentially helping to avoid unnecessary surgery. The small sample size is a major limitation, and these findings require validation in larger prospective studies.

**Conclusion:**

The combination of conventional ultrasound and CEUS may enhance the preoperative evaluation of renal hemangiomas. CEUS demonstrates distinctive enhancement patterns that can aid in differentiating these rare benign tumors from other renal malignancies. However, these findings are preliminary and require validation in larger-scale studies.

## 1 INTRODUCTION

Renal hemangioma is an extremely rare benign tumor that typically presents as an asymptomatic renal mass and is often discovered incidentally during imaging examinations [1, 2]. These benign tumors are characterized by a proliferation of blood vessels. Histologically, renal hemangiomas can be classified into various subtypes, including anastomosing hemangioma [3], which exhibits a distinctive pattern of anastomosing vascular channels and can mimic more aggressive tumors. Anastomosing hemangioma (AH) is an exceptionally rare benign vascular neoplasm, first characterized in 2009 and subsequently acknowledged by the World Health Organization (WHO) in its 2020 classification [4].The rarity of renal hemangioma results in a paucity of comprehensive imaging data, limiting the understanding of its imaging features and increasing the likelihood of misdiagnosis as malignancy [3, 4]. Recent literature underscores the importance of recognizing the distinct characteristics of renal hemangiomas and highlights the need for further research into their pathogenesis and optimal management [5]. Although renal hemangioma and renal angiomyolipoma share similar nomenclature, they represent distinct pathological entities. Renal hemangioma primarily comprises proliferating vascular endothelial cells, whereas angiomyolipoma is a mixed tumor composed of blood vessels, smooth muscle, and adipose tissue [6]. Furthermore, renal cell carcinoma differs from hemangioma in its imaging characteristics, clinical presentation, and management. Accurate differentiation between them is therefore crucial. Consequently, precise differentiation between the two is crucial for accurate diagnosis and effective management.

Due to their low incidence and potential overlapping imaging and histological features with other renal tumors, particularly renal cell carcinoma, diagnosing renal hemangiomas is challenging [7, 8]. The diagnosis of renal hemangiomas typically relies on imaging techniques such as computed tomography (CT) and magnetic resonance imaging (MRI) to differentiate them from malignant tumors6. This complexity makes accurate diagnosis challenging. The management of renal hemangiomas depends on tumor size and symptoms. Surgical intervention may be indicated for large or symptomatic tumors, while small, asymptomatic lesions can often be managed with active surveillance [9-11]. However, the prognosis is usually favorable. Therefore, investigating the application of new, accurate, and effective imaging techniques is essential to avoid misdiagnosis and overtreatment.

Contrast-enhanced ultrasound (CEUS) has undergone significant advancements in recent years. As a non-invasive imaging modality, CEUS has become instrumental in the diagnosis of hepatic and breast nodules, owing to its capacity for providing real-time dynamic observation [12-14]. Specifically, in the context of diagnosing hypoechoic hepatic hemangiomas, CEUS exhibits high diagnostic value, primarily characterized by peripheral nodule or ring enhancement, centripetal filling, and a rapid wash-in and slow wash-out phase transition [15]. Furthermore, CEUS is crucial in differentiating small atypical hepatocellular carcinoma from dysplastic nodules in patients with liver cirrhosis [16]. Moreover, contrast-enhanced ultrasound (CEUS) offers comprehensive insights into the vascular perfusion of breast nodules by enabling real-time observation of contrast agent distribution within the vessels [17]. This capability aids in differentiating between benign and malignant nodules. In the context of renal diseases, the 2018 guidelines from the European Federation of Societies for Ultrasound in Medicine and Biology (EFSUMB) highlighted the expanded utility of CEUS in the Bosniak classification of renal cystic lesions [18]. CEUS has demonstrated superiority over computed tomography (CT) in identifying thickened walls, septa, and solid components [19-21]. Consequently, as a radiation-free, cost-effective, and highly safe imaging modality, CEUS is anticipated to assume an increasingly significant role in the diagnosis, staging, and grading of renal diseases.

This study provides a comprehensive summary of ultrasound images from 20 patients diagnosed with renal hemangiomas, as confirmed by surgical pathology. A focused retrospective analysis was conducted on the ultrasound contrast imaging characteristics in a subset of these patients to describe preliminary imaging features to enhance the preoperative imaging diagnostic accuracy for these lesions.

## 2 MATERIALS AND METHODS

### 2.1 Study Subjects

We conducted a retrospective analysis of 20 cases of renal hemangiomas confirmed by surgical pathology between January 2018 and September 2024. All patients signed an informed consent form before surgery. Inclusion criteria were as follows:(1) patients with renal hemangiomas confirmed by surgical pathology and with complete clinical data; (2) patients who underwent routine ultrasound within one month before surgery. Six of these patients also underwent CEUS. (3) Patients who underwent contrast-enhanced computed tomography (CT) or magnetic resonance imaging (MRI) within one month before surgery. Exclusion criteria were: (1) poor-quality ultrasound images inadequate for clinical diagnosis; (2) incomplete CEUS examinations (*e.g*., missing arterial phase images). This study was conducted in accordance with the principles of the Declaration of Helsinki.

### 2.2 Examination Technique and Contrast Agent

Both CEUS and conventional ultrasound (CUS) examinations were performed using Philips EPIQ 7 (Philips Health Care, Andover, MA, USA), GE LOGIQ E9 (General Electric, Milwaukee, WI, USA), or SAMSUNG E9 ultrasound systems. The probe frequency utilized was approximately 1–6 MHz, and the mechanical index (MI) employed during CEUS scanning was approximately 0.08. Throughout the study period, all CEUS examinations were performed using a uniform CEUS imaging mode on Philips ultrasound systems, conducted by designated experienced physicians to ensure consistency in data acquisition. Before CEUS, the target lesion was identified on CUS. In cases with multiple lesions, the largest, best-visualized, and least respiration-affected lesion was selected for assessment. Ultrasound machine parameters, including depth, gain, and focus, were appropriately adjusted to optimize image quality. Upon initiation of CEUS, a bolus injection of 1.6 ml of the contrast agent (SonoVue, Bracco, Milan, Italy) was administered into the cubital vein, followed by a flush with 5 ml of 0.9% normal saline. The timing commenced upon the administration of the contrast agent. The target lesion was continuously monitored for a minimum duration of four minutes. All patients were instructed to breathe gently throughout the examination. In this study, the cortical phase was defined as 10–30 seconds post-injection, and the medullary phase was defined as starting at approximately 30 seconds post-injection and lasting until microbubble signals dissipated. A second injection was administered at least 20 minutes after the first, if clinically indicated. All static and dynamic images were captured using ultrasound machines.

The CUS and CEUS images were evaluated collaboratively by two radiologists (Y.C.H and H.M.N), each possessing over 10 years of experience in liver ultrasound examinations. These radiologists were blinded to the clinical data and pathological outcomes of all patients. The parameters assessed on CUS included the number of lesions, maximal lesion diameter, morphology, echogenicity, echogenicity consistency, presence of calcification, and boundary characteristics. The number of lesions was categorized as either single or multiple, while morphology was classified as regular or irregular.

Evaluations of Contrast-Enhanced Ultrasound (CEUS) encompassed several parameters, including the initial time to wash in, time to wash out, type of enhancement, velocity of enhancement, degree of enhancement relative to the surrounding normal renal parenchyma, consistency of enhancement, and presence of intratumoral necrosis. The onset of bubble formation was identified as the initiation of the entire enhancement process. We categorized the perfusion patterns into four distinct types: integral enhancement, characterized by an enhancement area encompassing more than 75% of the lesion; ring enhancement, defined as a peripheral enhancement area involving more than 50% of the lesion; rim enhancement, which refers to a peripheral enhancement area comprising less than 50% of the lesion; and dendritic enhancement, described as a strip or dendritic-shaped enhancement area within the lesion, based on the enhancement morphology observed during the arterial phase. The enhancement velocity was categorized into three groups: fast, synchronous, and slow enhancement. Additionally, the degree of enhancement was classified into hypo-enhancement and hyper-enhancement in comparison to the peripheral normal renal parenchyma at the peak enhancement time. Enhancement consistency was defined as either homogeneous or heterogeneous. Intratumoral necrosis was characterized by the presence of non-enhanced areas within the lesion, which was pathologically confirmed as necrosis.

### 2.3 Imaging Analysis

#### 2.3.1 Qualitative Analysis

The imaging data were independently evaluated by two ultrasound specialists who were blinded to the pathological outcomes and clinical details. In instances where their assessments diverged, a consensus was achieved through collaborative discussion. The evaluation criteria encompassed the anatomical location of renal tumors (cortex, medulla, or renal sinus), the maximum tumor diameter, morphological characteristics (round, oval, or irregular), echogenicity (including the presence of cystic changes, hemorrhage/thrombus, necrosis, or calcification), clarity of tumor boundaries, ultrasound image characteristics (hypoechoic, isoechoic, hyperechoic), and the enhancement features observed during ultrasound contrast imaging, specifically assessing whether the lesion enhancement in the delayed phase was homogeneous.

#### 2.3.2 Quantitative Analysis

The ultrasound machine was used to perform a quantitative analysis of the recorded video and ultrasound images obtained with ultrasound contrast. Specifically, the onset time of lesion enhancement and the time to peak enhancement were examined. Concurrently, the enhancement patterns of the ultrasound contrast were analyzed, including uniform enhancement, non-uniform enhancement, high enhancement, low enhancement, iso-enhancement, enhancement from the periphery to the center, and enhancement from the center to the periphery.

### 2.4 Pathological Analysis

Renal hemangiomas are pathologically classified into three distinct types: cavernous hemangioma, arteriovenous hemangioma, and capillary hemangioma. Histologically, these hemangiomas consist of dilated vascular structures characterized by irregularly arranged endothelial cells, exhibiting variable lumen sizes filled with red blood cells and small thrombi. The vessel walls are composed of fibroblasts and angioblasts. Immunohistochemical analysis reveals strong and diffuse staining for CD31 and/or CD34, with no evidence of mitotic activity in the nuclei, minimal or absent cellular atypia, and a low Ki-67 proliferation index. Pathologists categorize tumors according to the pathological characteristics of hemangiomas as follows: (1) Cavernous hemangioma, which consists of densely arranged large, thick-walled vessels, features a central spongy area and relatively sparse fibrous connective tissue; (2) anastomosing hemangiomas, characterized by the proliferation of dense capillaries and spike-like endothelial cells in an anastomotic pattern, with the interstitium potentially exhibiting edema and collagenous features; (3) Capillary hemangioma, primarily composed of fine vessels and extensive connective tissue, where endothelial cells are visibly spike-like, and the lesions typically measure less than 1 cm in diameter.

## 3 RESULTS

### 3.1 Clinical and Baseline Imaging Characteristics

This retrospective analysis included 20 patients with 22 pathologically confirmed renal hemangiomas. The demographic, clinical, and baseline lesion characteristics are summarized in Table **1**. The cohort comprised 10 men and 10 women, with a mean age of 50 years. Most tumors were discovered incidentally (14/20, 70.0%). Of the 22 lesions, 10 were in the right kidney and 12 in the left; 10 were cortical, and 12 were medullary or sinus in location. Two patients had multiple lesions, both of whom had end-stage renal disease (ESRD). The maximum tumor diameter ranged from 10 to 48 mm (mean, 27 ± 7 mm). Preoperative contrast-enhanced CT or MRI correctly suggested a vascular lesion in only 4 of 20 patients (20%). All patients underwent intervention: partial nephrectomy (n=8), radical nephrectomy (n=9), or transcatheter arterial embolization (n=3). During a median follow-up of 16 months (range, 6–70 months) in 18 patients, no local recurrence or metastasis was observed.

### 3.2 Conventional Ultrasound (CUS) Imaging Features

On conventional ultrasound, all lesions were solid masses. The majority (21/22, 95.5%) exhibited a regular round or oval shape. Most lesions (17/22, 77.3%) were hypoechoic. Internal echogenicity was homogeneous in 14 lesions (63.6%) and heterogeneous in 8 (36.4%); small cystic components were noted in 6 of the latter. Calcifications were rare (2/22, 9.1%). Color Doppler flow imaging (CDFI) detected intralesional or peripheral blood flow in 8 lesions (36.4%); the remaining 14 (63.6%) showed no significant flow. Only one lesion was associated with secondary hydronephrosis.

### 3.3 Contrast-enhanced Ultrasound (CEUS) Characteristics of Renal Hemangioma (n=6)

**Fig. (2):**
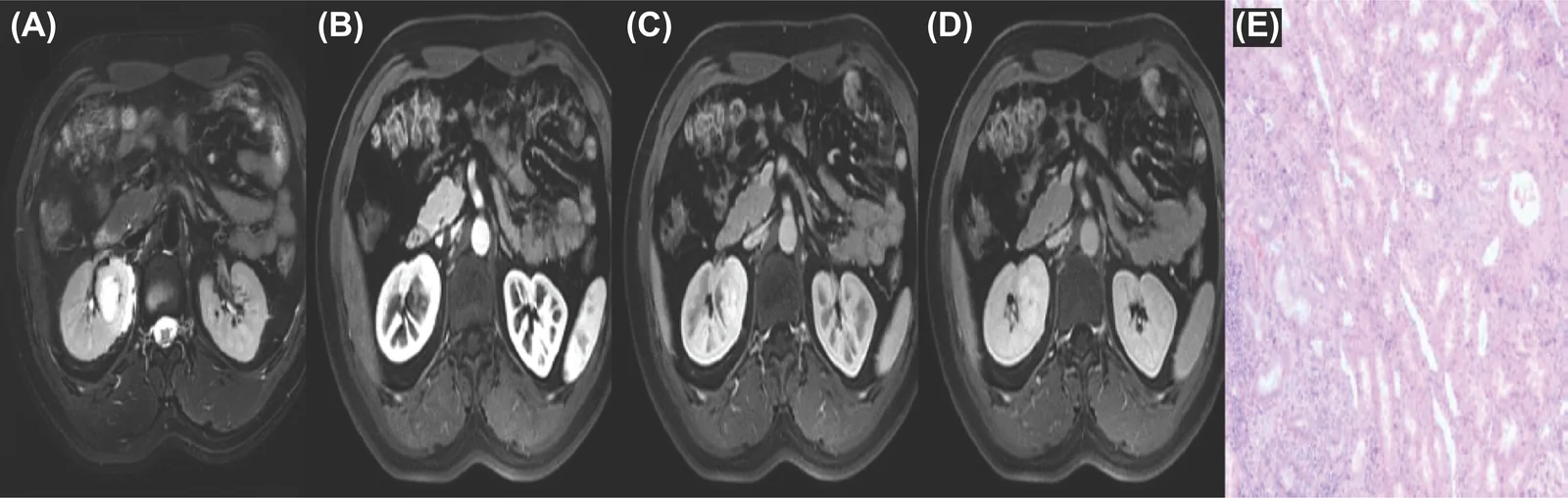
Patient Xu, a 28-year-old male. The MRI findings are as follows: **A**: T2-weighted imaging (T2WI) without contrast reveals a high signal intensity in the mass located in the right kidney. **B**: The contrast-enhanced scan shows nodular enhancement surrounding the right kidney mass during the corticomedullary phase, with an enhancement degree comparable to that of the renal cortex. **C**: During the parenchymal phase, the enhancement within the lesion progresses centripetally. **D**: In the excretory phase, the lesion in the right kidney exhibits a slightly delayed washout relative to the adjacent renal parenchyma. **E**: Hematoxylin and eosin (H-E) staining indicates the presence of vessels within the lesion that vary in thickness and size.

**Fig (4):**
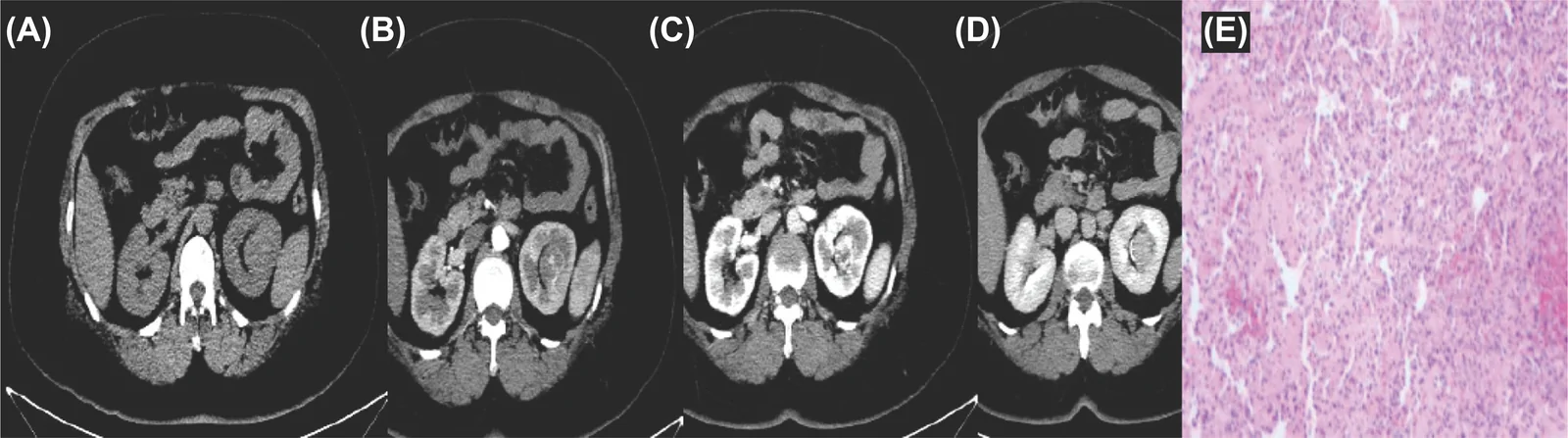
Patient Shen, a 50-year-old female. CT findings are as follows: **A**: Non-contrast scan shows a low-density lesion in the midportion of the left kidney. **B**: Contrast-enhanced scan demonstrates mild enhancement at the periphery of the left kidney mass during the parenchymal phase. **C**: During the medullary phase, the enhancement of the lesion fills from the periphery towards the center. **D**: In the excretory phase, the left kidney lesion shows a slightly earlier washout compared to the surrounding renal parenchyma. **E**: Hematoxylin and eosin (H-E) staining reveals diffuse anastomosing sinusoidal vascular spaces within the lesion.

In the study cohort, six patients (with a total of 7 lesions) underwent comprehensive contrast-enhanced ultrasound (CEUS) prior to surgical intervention. The CEUS features are detailed in Table **2**. Five patients had a solitary lesion. One patient had two medullary lesions in the right kidney and a history of chronic kidney disease (CKD). Among the five solitary lesions, four were hypoechoic on B-mode ultrasound (two with heterogeneous echotexture), and one was hyperechoic. On CEUS, these five nodules showed rapid peripheral hyperenhancement in the cortical phase, followed by progressive centripetal filling. Peak enhancement was reached in the medullary phase, with lesion enhancement remaining more pronounced than the adjacent renal parenchyma in the late phase. The patient with two lesions had stage IV chronic kidney disease (CKD) and was admitted due to hematuria. On B-mode ultrasound, both lesions were hypoechoic. The larger lesion (approximately 2.3 × 1.6 cm) had heterogeneous internal echotexture and no discernible blood flow on Color Doppler. No significant hydronephrosis was observed. Additionally, there was no significant hydronephrosis observed in the renal pelvis. As shown in Figs. (**1** and **3**), CEUS of the larger lesion demonstrated rapid peripheral enhancement in the cortical phase, followed by progressive centripetal filling. Peak enhancement was achieved in the medullary phase, with enhancement intensity remaining higher than the adjacent renal parenchyma in the late phase. Figures **2** and **4** are the MRI enhancement images of the lesions corresponding to Figs. (**1** and **3**) at different stages, as well as the final pathological results. Notably, the central region of the lesion consistently exhibited an absence of contrast agent entry throughout the entire process. The smaller lesion (approx. 0.7 × 0.4 cm), located near the renal sinus, had homogeneous internal echogenicity. On CEUS, it showed rapid hyperenhancement in the cortical phase, slightly exceeding the renal parenchyma. This was followed by washout in the medullary phase, becoming slightly hypoenhanced compared to the parenchyma.

Regarding enhancement patterns, five lesions showed ring-like enhancement, one showed peripheral enhancement, and one small lesion (0.7×0.4 cm) demonstrated immediate homogeneous enhancement. Among these lesions, five presented with heterogeneous enhancement, characterized predominantly by small regions within the masses that lacked contrast agent filling. The mean onset time for enhancement across the seven lesions was 12.2±3.1 seconds post-injection of the contrast agent, with an average peak enhancement time of approximately 26.9 ±5.4 seconds. Notably, only one lesion was hyperenhancing compared to the renal parenchyma; the remaining lesions were iso- or slightly hypoenhancing. Regarding washout patterns, four lesions exhibited synchronous washout with the surrounding renal parenchyma, and two demonstrated slightly delayed washout.

### 3.4 Pathological Features

Pathological Characteristics: Among the cohort of 20 patients, pathological examination revealed 9 instances of anastomosing hemangioma, 2 instances of cavernous hemangioma, and 9 instances of capillary hemangioma. Comprehensive immunohistochemical analysis was conducted on tumor samples from all patients, revealing an absence of significant mitotic figures or nuclear atypia. The immunohistochemical profile demonstrated positivity for CD31, CD34, and Ki-67, although Ki-67 activity was observed to be less than 10% across all cases. Notably, 5 patients had a documented history of chronic kidney disease, with 2 of these individuals presenting with multiple lesions, all identified as capillary hemangiomas.

## 4 DISCUSSION

### 4.1 Study Rationale and Context

Renal hemangiomas are rare, benign vascular tumors whose diagnosis is challenging due to their low incidence and overlapping imaging features with malignant renal tumors, particularly renal cell carcinoma [22-24]. While conventional ultrasound features have been sporadically reported, systematic descriptions of their contrast-enhanced ultrasound (CEUS) characteristics a modality that visualizes real-time microvascular perfusion are exceedingly scarce [24, 25]. This knowledge gap directly impacts preoperative diagnosis. As evidenced by our and other series, misdiagnosis rates based on conventional cross-sectional imaging (CT/MRI) remain high [26-28].Therefore, this study aimed to address this deficiency by providing a structured analysis of CEUS features in a pathologically confirmed cohort and to preliminarily explore the diagnostic potential of CEUS for this rare entity.

### 4.2 Clinical and Conventional Ultrasound Features

Our demographic findings including equal gender distribution, a median age of 50 years, and a mean tumor diameter of 2.6 cm are consistent with prior reports [29]. Most tumors were incidental findings, and symptoms were uncommon, consistent with the indolent nature of these tumors [30]. The predominance of lesions in the renal medulla and left kidney in our series, while noted, requires further investigation in larger populations.

Conventional ultrasound typically revealed well-defined, hypoechoic, round masses, findings that are non-specific and often mimic renal cell carcinoma [31, 32]. Color Doppler flow imaging (CDFI) had limited utility, detecting flow signals in only 36% of lesions. This is likely attributable to the slow flow within the tumor's variably sized vessels [33]. This underscores the inherent limitation of relying solely on conventional ultrasound for diagnosis.

### 4.3 Pathological Correlation

Histopathologically, anastomosing hemangioma was the most prevalent subtype in our cohort, followed by capillary hemangioma a distribution supported by recent literature [34]. The association between hemangiomas (particularly multiple lesions) and end-stage renal disease (ESRD) observed here has been previously reported [29, 35]. The underlying histoarchitecture comprising dilated, tortuous vascular channels explains the key imaging observations: frequent hypoechogenicity, limited Doppler flow detection, and, crucially, the characteristic CEUS enhancement patterns detailed below.

### 4.4 Contrast-Enhanced Ultrasound (CEUS) Features

Our analysis of 7 lesions in 6 patients delineates preliminary CEUS patterns of renal hemangiomas. The most characteristic pattern observed (5/7 lesions) was peripheral nodular enhancement in the cortical phase with progressive centripetal filling, analogous to the hallmark of hepatic hemangiomas [36, 37]. This pattern corresponds to the slow, peripheral-to-central perfusion of the tumor's vascular network.

Peak enhancement intensity was typically comparable to (iso-enhancing) or slightly lower than (hypoenhancing) the renal parenchyma, with synchronous or slightly delayed washout. This differs from hepatic hemangiomas, which typically exhibit stronger and more persistent enhancement. The reasons for this difference are likely multifactorial. While the anatomical difference in organ perfusion (renal single vs. hepatic dual blood supply) may contribute, organ-specific microvascular flow dynamics and differences in vascular endothelial biology between these benign tumors warrant further investigation. Heterogeneous enhancement was common (5/7 lesions), attributable to intralesional thrombosis, fibrosis, or cystic changes as seen in one of our cases and corroborated by other reports [37, 38]. The single small lesion demonstrating immediate homogeneous fill highlights how size can significantly influence the perceived enhancement pattern.

### 4.5 Differential Diagnosis: CEUS Comparison with Common Renal Tumors

The observed CEUS patterns aid in differentiating renal hemangiomas from other common solid renal masses, notably renal cell carcinoma (RCC) and lipid-poor angiomyolipoma (lp-AML).

Versus Renal Cell Carcinoma: Clear cell RCC typically displays rapid, marked, and homogeneous hyperenhancement (“fast-in and fast-out”) reflecting its rich neovascularization [37]. In contrast, our cases showed a slower, peripheral-to-central filling pattern with iso- to mild hypoenhancement at peak (“slow-in and synchronous-out”). Papillary RCC, often consistently hypo-enhancing, also differs from this progressive filling pattern.

Versus Lipid-Poor Angiomyolipoma: While both may appear solid without fat on conventional imaging, LP-AML on CEUS usually lacks the stereotypical peripheral nodular enhancement with centripetal progression seen in most of our hemangiomas. Its enhancement tends to be more heterogeneous and diffuse from the arterial phase [39, 40].

Synthesis: The slow, peripheral-to-central filling of renal hemangioma contrasts with the rapid, diffuse enhancement of clear cell RCC and the more heterogeneous enhancement of lp-AML. Recognizing this CEUS signature can help steer diagnostic suspicion toward a benign vascular etiology.

### 4.6 Diagnostic Implications and Synthesis

In synthesis, renal hemangiomas in our series were most frequently medullary-based anastomosing hemangiomas, presenting on ultrasound as well-defined hypoechoic masses with scant Doppler flow. CEUS adds a critical dynamic dimension, often showing slow, peripheral-to-central filling with iso-enhancement at peak. Integrating these features especially the CEUS pattern can raise suspicion for a benign vascular lesion, aiding in its differentiation from renal cell carcinoma, which typically exhibits different enhancement kinetics.

However, when imaging features are atypical (*e.g*., due to hemorrhage, thrombosis, or small lesion size), the risk of misdiagnosis remains. Therefore, while CEUS can provide valuable diagnostic clues and may help avoid unnecessary surgery, pathological confirmation *via* biopsy remains the gold standard, particularly in diagnostically uncertain cases.

### 4.7 Limitations and Future Perspectives

Our study has several limitations that should be considered when interpreting the findings on contrast-enhanced ultrasound (CEUS) renal hemangioma. The most notable limitation is the small sample size of the CEUS cohort (6 patients with 7 lesions), which reflects the inherent challenge of studying this rare condition in a single-center, retrospective setting. This limits the statistical power of our analysis and the generalizability of the observed CEUS features. Therefore, the CEUS characteristics described here should be viewed as preliminary and descriptive, serving as a basis for hypothesis generation. Additionally, the retrospective design may introduce selection and information biases.

Future prospective, multi-center studies with larger cohorts are needed to validate and refine these CEUS features, establish diagnostic criteria, and evaluate their performance against other imaging modalities. Further investigation into quantitative CEUS parameters may help identify objective diagnostic markers. Correlating specific imaging patterns with histopathological subtypes could further elucidate the disease and its imaging manifestations.

## CONCLUSION

This study provides a comprehensive analysis of the ultrasound imaging characteristics of renal hemangiomas, with a focus on the preliminary role of contrast-enhanced ultrasound (CEUS) in diagnosing these rare benign tumors. Our findings indicate that renal hemangiomas predominantly occur in the renal medulla, with anastomosing hemangioma being the most common subtype. On conventional ultrasound, they typically appear as well-defined, hypoechoic, round masses. In contrast, preliminary CEUS observations reveal a distinctive pattern of peripheral nodular enhancement in the arterial phase with progressive centripetal filling, reaching peak intensity that is generally comparable to the renal parenchyma. The integration of conventional ultrasound with CEUS may improve preoperative diagnostic evaluation, offering substantial practical value in guiding clinical management. This combined approach could help differentiate renal hemangiomas from other renal tumors, potentially reducing the risk of misdiagnosis and unnecessary interventions.

In conclusion, this study highlights the potential of CEUS as a valuable diagnostic modality for renal hemangiomas, demonstrating its ability to provide detailed imaging characteristics that may improve diagnostic accuracy and inform management. Further validation in larger studies is warranted.

## Figures and Tables

**Fig. (1):**
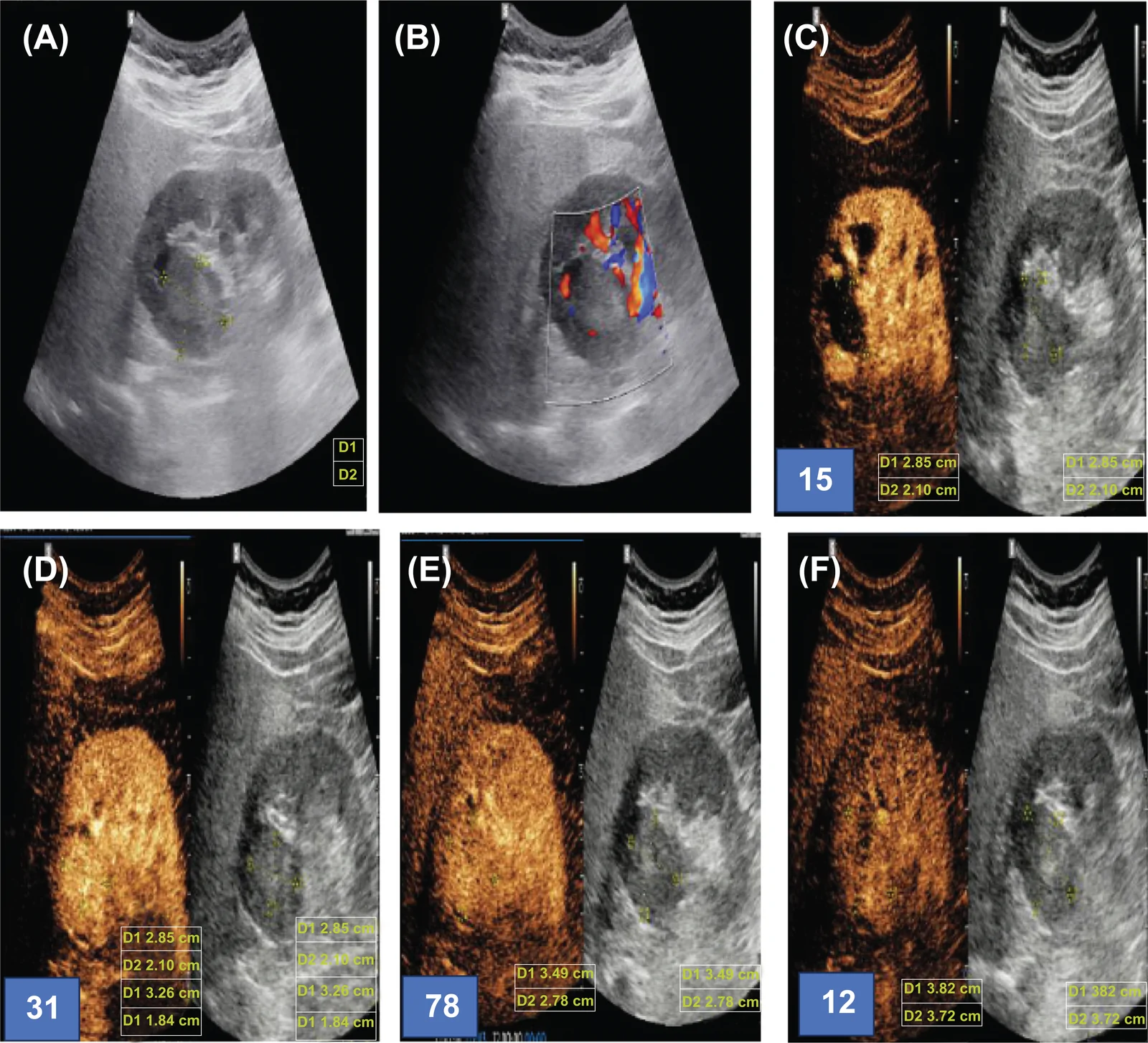
Patient Xu, a 28-year-old male. **A**: Two-dimensional ultrasound shows a lesion ultrasound examination which revealed a lesion situated within the renal pelvis of the right kidney. The lesion measures approximately 3.8 × 2.8 cm, exhibits an oval shape, and possesses relatively well-defined margins. No significant hydronephrosis was observed in the collecting system of the right kidney. **B**: Color Doppler Flow Imaging (CDFI) demonstrated a star-like blood flow signal surrounding the lesion, with sparse blood flow within the lesion itself. **C**: Upon administration of the SonoVue ultrasound contrast agent, the timing of enhancement was noted. At 15 seconds post-injection, the cortex of the right kidney was fully enhanced, whereas the lesion began to enhance at a slower rate. **D**: Between 18 and 31 seconds, the contrast agent gradually enhanced the lesion from its periphery to its interior. By 31 seconds, the lesion was completely filled with the contrast agent, achieving an enhancement level nearly equivalent to that of the renal cortex. **E**: At 78 seconds, the contrast agent in the renal cortex began to dissipate slowly, at which point the lesion appeared slightly more enhanced than the surrounding renal parenchyma. **F**: At 120 seconds, the contrast agent in the renal cortex further fades, and the lesion in the right kidney fades slightly later than the surrounding renal parenchyma.

**Fig. (3):**
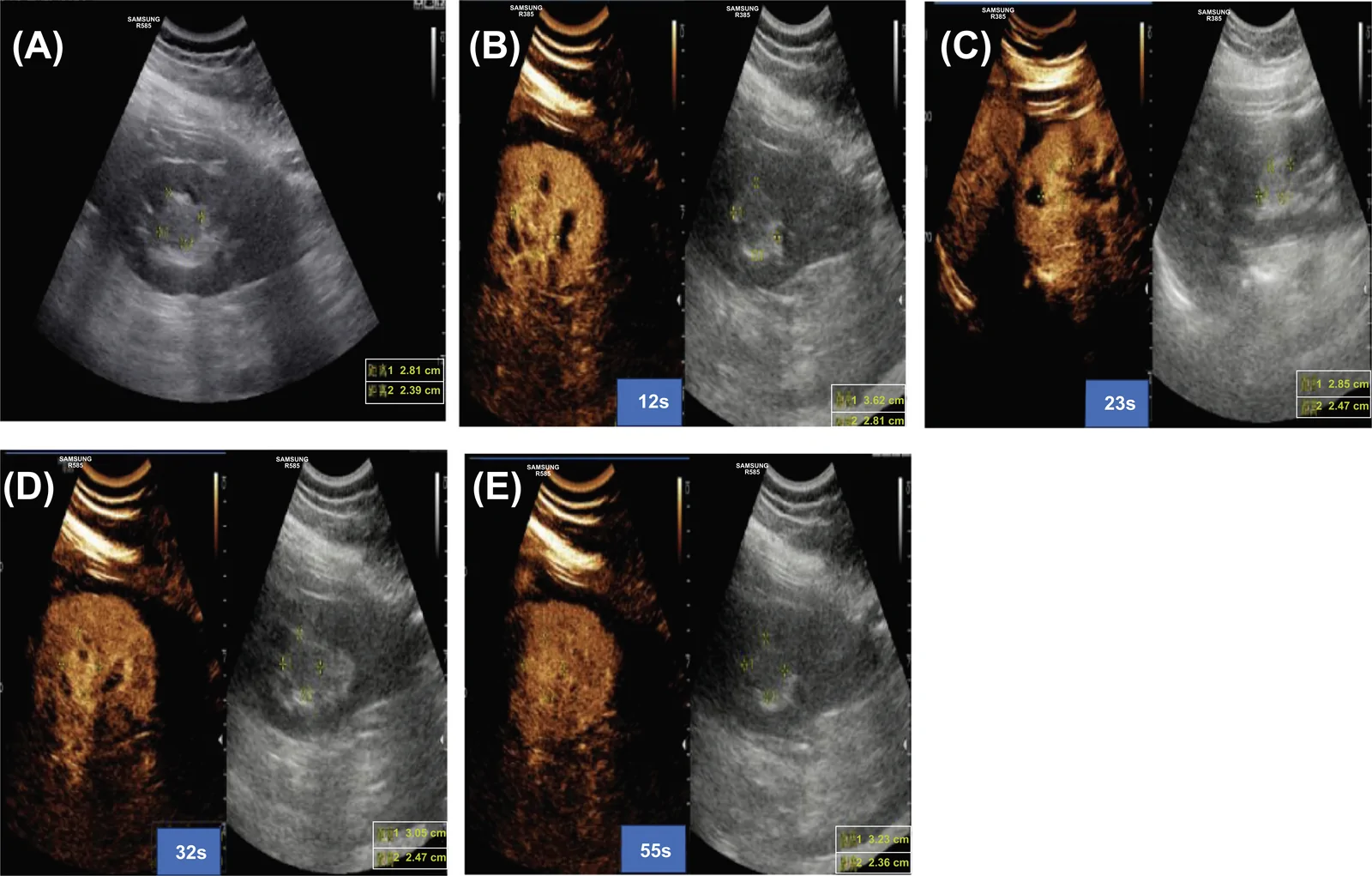
Patient Shen, a 50-year-old female. **A**: Two-dimensional ultrasound shows that the lesion is located within the left renal sinus, presenting as hypoechoic, with a size of approximately 3 × 2.7 cm, oval-shaped, and with relatively clear margins. **B**: At 12 seconds after bolus injection of SonoVue, the lesion shows peripheral nodular enhancement, the lesion in the left kidney shows nodular enhancement at the periphery during the cortical phase. **C**: At 23 seconds, the contrast agent enhances slowly from the periphery towards the center of the lesion. **D**: At 32 seconds, the contrast agent has completely filled the lesion, and at this time, the degree of enhancement of the lesion is slightly higher than that of the surrounding renal cortex. **E**: At 55 seconds, the contrast agent in the renal cortex begins to slowly fade, and at this time, the lesion in the left kidney appears slightly higher than the surrounding renal parenchyma.

**Table 1:** Clinical and baseline characteristics of 20 patients with renal hemangioma.

**Characteristic**	**Value**
Patients, n	20
Lesions, n	22
Age (years), mean	50
Sex, n (%)	
Male	10 (50.0)
Female	10 (50.0)
Mode of Discovery, n (%)	
Incidental (physical exam/dialysis check)	14 (70.0)
Symptomatic (hematuria/flank pain)	6 (30.0)
Lesion Laterality (n=22), n (%)	
Right Kidney	10 (45.5)
Left Kidney	12 (54.5)
Lesion Location (n=22), n (%)	
Cortex	10 (45.5)
Medulla / Sinus	12 (54.5)
Multiplicity, n (%)	
Single Lesion	18 (90.0)
Multiple Lesions	2 (10.0)
Max Diameter (mm), mean ± SD	27 ± 7
History of CKD (Stage V), n (%)	5 (25.0)
Preop CT/MRI Diagnosis Correct	4 (20.0)

**Table 2:** The ultrasound contrast enhancement (CEUS) findings of seven lesions in six patients.

**Patient ID**	**Lesion Size** **(cm)**	**Enhancement Pattern**	**Internal Homogeneity**	**Enhancement Speed**	**Enhancement Degree**	**Start of Enhancement Time (s)**	**Peak Enhancement Time** **(s)**	**Washout**	**Pathology**
1	3.8×2.8	Ring enhancement	Homogeneous	Slower	Equal	15	31	Later	Cavernous hemangioma
2	3×2.7	Ring enhancement	Heterogeneous	Slower	Strong	12	27	Later	Anastomosing hemangioma
3	4.8×4	Ring enhancement	Heterogeneous	Slower	Equal	16	33	Concurrent	Cavernous hemangioma
4	2.3×1.2	Ring enhancement	Heterogeneous	Slower	Low	12	21	Concurrent	Capillary hemangioma
5	1.9×1.6	Marginal enhancement	Heterogeneous	Slower	Equal	12	34	Concurrent	Capillary hemangioma
6	2.3×1.6	Ring enhancement	Heterogeneous	Slower	Equal	12	22	Concurrent	Capillary hemangioma
7	0.7×0.4	Homogeneous enhancement	Homogeneous	Slower	Strong	10	20	Slightly earlier	Capillary hemangioma

## Data Availability

The data and supportive information are available within the article.

## LIST OF ABBREVIATIONS

CEUS: Contrast-enhanced ultrasound
AH: Anastomosing hemangioma
WHO: World Health Organization
CT: Computed tomography
MRI: Magnetic resonance imaging

## STANDARDS OF REPORTING

STROBE guidelines were followed.
